# Geographical distribution of family physicians in Japan: a nationwide cross-sectional study

**DOI:** 10.1186/s12875-019-1040-6

**Published:** 2019-10-29

**Authors:** Shuhei Yoshida, Masatoshi Matsumoto, Saori Kashima, Soichi Koike, Susumu Tazuma, Takahiro Maeda

**Affiliations:** 1 Department of Community-Based Medical Systems, Graduate School of Biomedical and Health Sciences, Hiroshima University, 1-2-3 Kasumi, Minami-ku, Hiroshima, 734-8551 Japan; 2 Environmental Health Sciences Laboratory, Department of Development Technology, Graduate School for International Development and Cooperation, Hiroshima University, Higashi-Hiroshima, Japan; 3 Division of Health Policy and Management, Center for Community Medicine, Jichi Medical University, 3311-1 Yakushiji, Shimotsuke, Tochigi, 329-0498 Japan; 4 Department of General Internal Medicine, Hiroshima University Hospital and Graduate School of Biomedical and Health Sciences, Hiroshima, Japan; 5 Department of Community Medicine, Nagasaki University Graduate School of Biomedical Science, Nagasaki, Japan

**Keywords:** Geography, Health policy, Japan, Family physician

## Abstract

**Background:**

Geographical maldistribution of physicians, and their subsequent shortage in rural areas, has been a serious problem in Japan and in other countries. Family Medicine, a new board-certified specialty started 10 years ago in Japan by Japan Primary Care Association (JPCA), may be a solution to this problem.

**Methods:**

We obtained the workplace information of 527 (78.4%) of the 672 JPCA-certified family physicians from an online database. From the national census data, we also obtained the workplace information of board-certified general internists, surgeons, obstetricians/gynaecologists and paediatricians and of all physicians as the same-generation comparison group (ages 30 to 49). Chi-squared test and residual analysis were conducted to compare the distribution between family physicians and other specialists.

**Results:**

Five hundred nineteen JPCA-certified family physicians and 137,587 same-generation physicians were analysed. The distribution of family physicians was skewed to municipalities with a lower population density, which shows a sharp contrast to the urban-biased distribution of other specialists. The proportion of family physicians in non-metropolitan municipalities was significantly higher than that expected based on the distribution of all same-generation physicians (*p* < 0.001).

**Conclusions:**

Family physicians distributed in favour of rural areas much more than any other specialists in Japan. The better balance of family physician distribution reported from countries with a strong primary care orientation seems to hold even in a country where primary care orientation is weak, physician distribution is not regulated, and patients have free access to healthcare. Family physicians comprise only 0.2% of all Japanese physicians. However, if their population grows, they can potentially rectify the imbalance of physician distribution. Government support is mandatory to promote family medicine in Japan.

## Background

The geographical maldistribution of physicians, and their subsequent shortage in rural areas, is a serious social problem in Japan and internationally [1]. The total population of Japan is 127 million [2]. People 65 years of age or older account for 28.1%. The vast majority of the population (91.4%) lives in urban areas. The dearth of physicians in rural areas has persisted in Japan despite various large-scale political interventions by the government such as the ‘one medical school in each prefecture policy’ in 1970s and 1980s and foundation of a special medical school, Jichi Medical University, for producing rural physicians in 1972 [3–5]**.** This maldistribution has even worsened since 2004 when the new residency training programme for postgraduate year 1 and year 2 physicians was implemented nationwide [6, 7].

Primary care physicians reportedly distribute better than other physicians in some countries with a strong primary care orientation, but it is unknown whether this is the case in a country like Japan with a weak primary care orientation, no regulation of physician distribution, and patients’ free access to physicians [8, 9].

Family medicine has not traditionally been recognised as an independent academic discipline in Japan. Primary care was provided by other specialists such as internists and paediatricians [10]. They were trained in a hospital setting as a hospital-based specialist, and then many of them gradually moved into clinic-based primary care physician as a second career, without re-education as a generalist. This movement from a hospital to a clinic is because clinic-based physicians generally earn more than hospital-based physicians. The transition from a specialist to a generalist is facilitated by absence of a formal training system to produce family physicians in Japan [11]. Under these circumstances, to improve the quality of primary care, the Japan Primary Care Association (JPCA) introduced board certification as the only qualification for primary care physicians in the country in 2009 [12]. However, family medicine remains unpopular in Japan. The total number of JPCA-certified family physicians was only 672 (0.2%) of 311,205 physicians in 2018 [13].

Then a new board-certification system by the Japanese Medical Specialty Board, which is independent of existing specialist bodies, started from 2018 and ‘general practice’ was added as the 19th major clinical discipline. The certification of family physicians/general practitioners thus will undergo transition from JPCA to the Japanese Medical Specialty Board in 2021. With the reform of board certification, the number of trained general practitioners is expected to increase. In actuality, however, of the 8217 applicants to specialist training programs in 2019, only 158 (1.9%) applied to ‘general practice’ [14]. This means that the minority status of primary care physicians in Japan will remain largely unchanged. In Japan’s medical fee system, the salaries of physicians employed by hospitals are based largely on their career length and administrative position. So their incomes among specialties do not differ substantially. However self-employed physicians, most of whom are based in their own clinics and provide primary care, earn, on average, as much as twice the salary of employed physicians regardless of specialty. But the very high initial cost to set up a private clinic is the largest barrier for early-career physicians to become self-employed. So early career physicians in Japan usually work as hospital-based specialists for more than 10 yrs and then become clinic-based primary care physicians [15]. There is no governmental regulation on the movement from a hospital to clinic or from a specialist to generalist. Because the board-certification of family medicine or general practice is not a requirement for being a self-employed primary care physician, there is little financial incentive to be board-certified in Japan.

Japan’s national and local governments have very limited amount of authority to regulate the distribution of physicians or cap the number of physicians in cities. Consequently, physicians, regardless of their specialties, tend to concentrate in urban areas. Moreover, Japanese patients can choose any physician that they like, which encourages the movement of patients from rural to urban areas. This makes Japan an ideal place to examine the distribution of family physicians. If Japanese family physicians turn out to distribute better than other physicians, the fairness of the distribution would be more in the nature of family medicine than in that of the healthcare or economic system that regulates the distribution of primary care physicians.

In this study we evaluated the geographic distribution of Japanese family physicians and compared it with same-generation physicians of other specialties. We then discussed the potential effectiveness of promotion of family medicine in Japan for the more equal access of its population to healthcare.

## Methods

### Study setting

The setting of this study is nationwide, which includes all the 47 prefectures of Japan.

The definitions of “family physicians”, “general practitioners” and “primary care physicians”.

Family physicians are qualified as such by JPCA: *katei-iryou-senmoni*. In the new specialist training scheme, general practitioners will be certified as such by the Japanese Medical Specialty Board: *sougou-sinryou-senmoni*. In other words, general practitioners are future generalists that will succeed JPCA family physicians. Primary care physicians are defined as all physicians who offer primary care regardless of their certifications. This group includes family physicians, general practitioners and other specialists who become primary care providers as their second career.

### Data of JPCA-certified family physicians

We used an online database, current as of 31 July 2018, of 527 (78.4%) of the 672 JPCA-certified family physicians who gave the permission to the JPCA office [13]. The website discloses each physician’s name, workplace (municipality and medical institution) and area of interest.

### Data on other physicians in Japan

To estimate the age of the certified family physicians, we searched the year of physician license registration of each JPCA-certified family physician, using the open online database of the Ministry of Health, Labour and Welfare [16]. We found the registration year of 467 (88.6%) physicians. As a result, their median (interquartile range) age in 2018 was estimated to be 37 (31–48). We extracted, as the comparison group of the family physicians, physicians between the ages of 30 and 49 from all physicians in the dataset of the ‘Survey of Physicians, Dentists and Pharmacists’ (Physician Census) conducted by the Ministry of Health, Labour and Welfare in December 2016. The Census contained information on the major board-certification for all registered physicians in Japan, but the information on JPCA certification was not included because family medicine was not recognised as a formal clinical disciple until 2018. Within the comparison group we identified all board-certified general internists (Fellow of the Japanese Society of Internal Medicine: *sougou-naika-senmoni*), surgeons (Japan Surgical Society), obstetricians/gynaecologists (Japan Society of Obstetrics and Gynecology), and paediatricians (Japan Pediatric Society) in the Census. Individual data in the Census was used with special permission of the Ministry (permission no. 0411–3).

### Data on municipalities

Japan has three levels of government: national, prefectural and municipal. Municipalities comprise cities, towns and villages. We compared the rurality of workplace municipality of family physicians with that of the comparison group physicians. Using the data on population and land area in each municipality published by the Statistics Bureau, Ministry of Internal Affairs and Communications [17], we divided municipalities into quintiles sorted by population density (Quintile 1 < = 433.14, Quintile 2 < = 1195.59, Quintile 3 < = 3155.03, Quintile 4 < = 10,845.34, Quintile 510,845.34+ people per square kilometer) so that each quintile has 20% of all municipalities. To measure rurality in another way, we divided municipalities into ‘metropolis,’ ‘city’ and ‘town/village.’ ‘Metropolis’ was all of the ordinance-designated cities (seirei-shitei-toshi) and 23 special wards of Tokyo (*n* = 171). ‘City’ was the other cities (shi) (*n* = 756). ‘Town/village’ was towns (cho) and villages (son) (*n* = 884).

### Statistical analysis

A chi-square test was conducted to compare the distribution of categorical data between two groups. Residual analysis was used to compare the real value and the expected value derived from the distribution of all physicians. All statistical analyses were performed using Microsoft Excel and STATA/SE version 15 (StataCorp, 2017).

## Results

Eight family physicians were excluded due to lack of precise workplace information, leaving a total of 519 family physicians and 137,587 same-generation physicians (including 11,947 general internists, 11,570 surgeons, 5268 obstetricians/gynaecologists, and 6919 paediatricians).

Table 1 shows the distribution of each group of physicians among the quintiles of municipalities sorted by population density. The proportion of family physicians in municipalities with a low population density was significantly higher than the proportion expected based on the distribution of all same-generation physicians. General internists and surgeons distributed almost similarly to all the physicians, and obstetricians/gynaecologists and paediatricians distributed slightly more to urban areas than all physicians.

**Table 1:** Workplace municipalities of board-certified physicians ages 30 to 49, classified by population density

		Quintile of municipalities sorted by population density^a^					Total
		1	2	3	4	5	
Family physicians	N	27**^+^	49**^+^	75**^+^	138**^+^	230**^−^	519
	%	5.2%	9.4%	14.5%	26.6%	44.3%	100.0%
General internists	N	62	271	873	2599	8142	11,947
	%	0.5%	2.3%	7.3%	21.8%	68.1%	100.0%
Surgeons	N	51	302	894	26009*^+^	7723**^−^	11,570
	%	0.4%	2.6%	7.7%	22.5%	66.8%	100.0%
Obstetricians and gynaecologists	N	7**^−^	121	318**^−^	1085	1435**^+^	5268
	%	0.1%	2.3%	6.0%	20.6%	70.9%	100.0%
Paediatricians	N	17**^−^	148	545	1435*^−^	4774*^+^	6919
	%	0.2	2.1	7.9	20.7	69.0	100.0%
All physicians^b^	N	747	3846	10,830	29,489	92,675	137,587
	%	0.5	2.8	7.9	21.4	67.4	100.0%

^a^Quintile 1 < = 433.14, Quintile 2 < = 1195.59, Quintile 3 < = 3155.03, Quintile 4 < = 10,845.34, Quintile 510,845.34+ people per square kilometer

^b^All physicians were not included in the residual analysis

Residual analysis based on chi square test which examines the difference between the real and expected value at each cell.

*^+^: greater than expected value. *P* < 0.05

**^+^: greater than expected value. *P* < 0.001

*^−^: less than expected value. P < 0.05

**^−^: less than expected value. P < 0.001

**Fig. 1:**
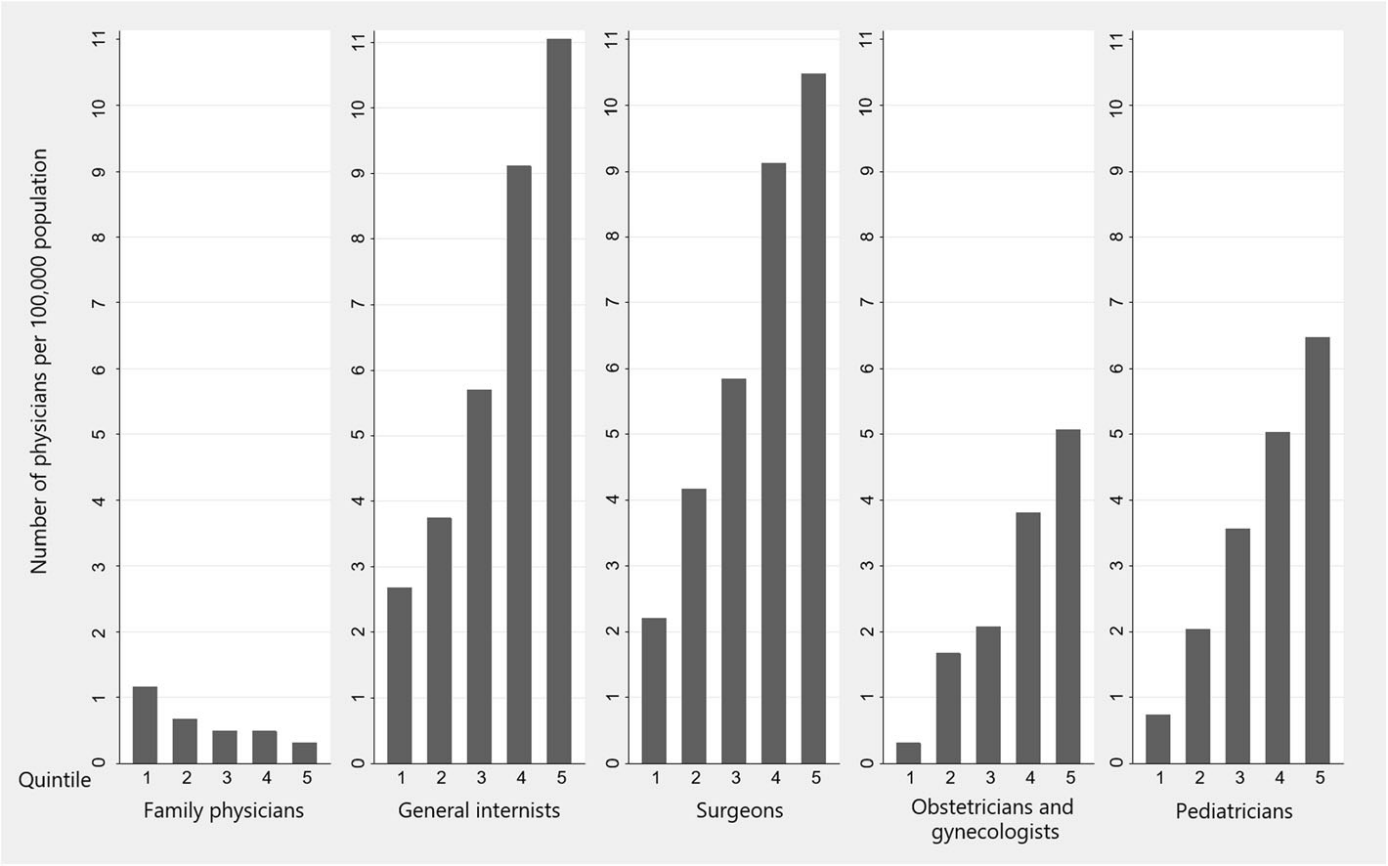
Number of board-certified physicians ages 30 to 49 per 100,000 population, classified by population density

Figure 1 shows the number of board-certified physicians per 100,000 population classified by population density. The population of family physicians was tiny, but their distribution was skewed more to rural areas than to urban areas, which is quite contrary to the urban-biased distribution of physicians in other specialties.

Table 2 shows the distribution of each group of physicians among the three municipality types. The proportion of family physicians in rural municipalities was significantly higher than expected based on the distribution of all same-generation physicians. The distributions of physicians in other specialties were not largely different from the distribution of all physicians.

**Table 2:** Workplace municipalities of board-certified physicians ages 30 to 49, classified by municipality type

		Municipality type ^a^			Total
		Metropolis^a^	City^b^	Town/village^c^	
Family physicians	N	194**^−^	260**^+^	65**^+^	519
	%	37.4%	50.1%	12.5%	100.0%
General internists	N	7299	4194	454	11,947
	%	61.1%	35.1%	3.8%	100.0%
Surgeons	N	6928	4167	475	11,570
	%	59.9%	36.0%	4.1%	100.0%
Obstetricians and gynaecologists	N	3299**^−^	1811*^−^	158**^−^	5268
	%	62.6%	34.4%	3.0%	100.0%
Paediatricians	N	4164	2493	262	6919
	%	60.2	36.0	3.8	100.0%
All physicians^d^	N	81,794	49,858	5935	137,587
	%	59.4	36.2	4.3	100.0%

^a^ Metropolis: all the ordinance-designated cities (seirei-shitei-toshi and chukaku-shi) and 23 special wards of Tokyo (ku) (*n* = 97)

^b^City: the other cities (shi) (*n* = 717)

^c^Town/village: towns (cho) and villages (son) (*n* = 923)

^d^All physicians were not included in the residual analysis

Residual analysis based on chi square test which examines the difference between the real and expected value at each cell.

*^+^: greater than expected value. *P* < 0.05

**^+^: greater than expected value. *P* < 0.001

*^−^: less than expected value. *P* < 0.05

**^−^: less than expected value. *P* < 0.001

**Fig. 2:**
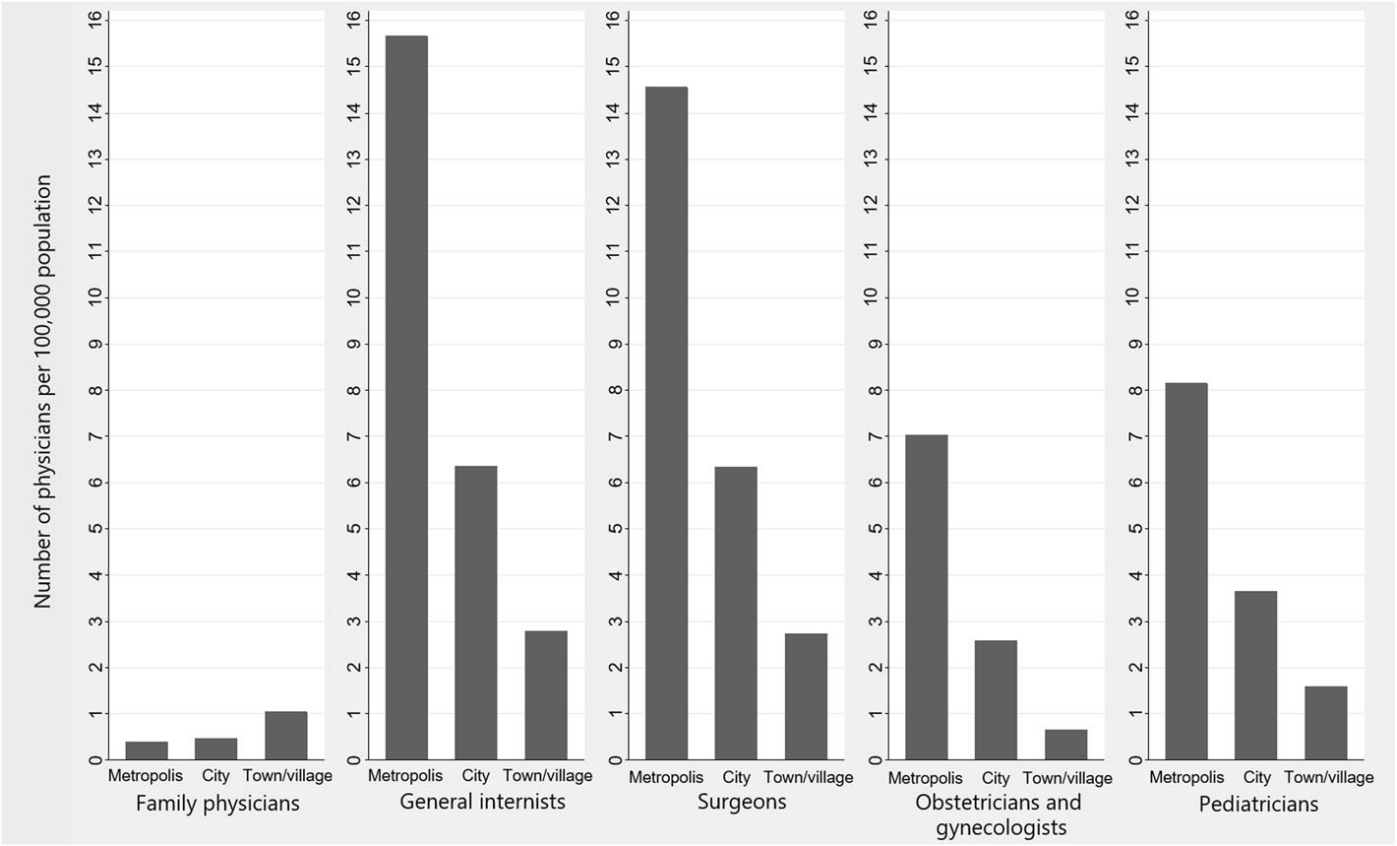
Number of board-certified physicians ages 30 to 49 per 100,000 population, classified by municipality type

Figure 2 shows the number of board-certified physicians per 100,000 population classified by municipality types. As with the population density, the distribution of family physicians was much more rural-biased than the distributions of other specialties although the population of family physicians was tiny.

## Discussion

Although the total number of family physicians was much lower than those of other physicians, family physicians in Japan distributed more to rural than to urban areas, in stark contrast to the urban-biased distribution of other physicians. This study suggests family physicians have a very favourable distribution even in a country with a poor primary care orientation, no regulation on the distribution of physicians, and free access of people to physicians.

The better distribution of general practitioners and urban-biased distribution of other specialists were also observed in Europe [18, 19]. In countries with a strong primary care orientation and a public sector-based healthcare system such as the United Kingdom and the Netherlands, everyone is required to register with his/her general practitioner. It is quite reasonable, in such countries, for general practitioners to distribute according to population. However, the Japanese healthcare system is unique. The Japanese do not need to register with a certain primary care physician. Their access to any physician is not limited by insurance companies or the government. All the Japanese are covered by public health insurance and they can choose their physicians of any specialty. In addition, physicians can choose where to practice without any governmental regulation. This study showed, even in Japan, family physicians distributed quite equally, or even disproportionately to rural areas.

Accessibility, both financial and geographical, is a principle of primary care [20]. To ensure the quality of primary care, physicians need to ensure equal and good accessibility to their patients [21]. The egalitarian principle of primary care might help family physicians to voluntarily distribute equally or more to places where they are in greatest demand.

Another potential reason for a rural-biased distribution of Japanese family physicians is the cost of practicing in urban areas, especially for young family physicians. In Japan, most clinics are managed by an individual or a family. A relatively few physicians are hired to work in clinics. The initial cost to establish a clinic is estimated, on average, at 94 million yen (758,000 euro), 20 million of which come from the physician’s own savings [22]. In Japan, early-career physicians are usually hired by a hospital and then open their own clinics in the middle or towards the end of their career. The median age of our study participants was 37. Because early-career physicians do not have 20 million yen in savings, if they become family physicians, they may be limited to working in public clinics established by municipalities in areas with a physician shortage. This might tilt the distribution of Japanese family physicians towards rural areas.

The results of this study suggest that the increase of family physicians might rectify the present urban-skewed distribution of physicians, which has long been a serious social problem in Japan [4]. However, the number of Japanese family physicians was negligible, because of the short history of JPCA certification and the lack of governmental support for increasing the population of family physicians. A small but important first step to the expansion of this group of physicians was the introduction of ‘general practice’ as one of 19 major clinical disciplines under the new training system for board certification starting in 2018. However, against expectations, the number of applicants to ‘general practice’ was only 1774 (2.1%) of the 8604 applicants in 2019. The low popularity is probably caused by low awareness among population and lack of governmental political support for general practice in Japan [23].

Family physicians are pivotal in providing care in Japan where the population is rapidly ageing and more patients than ever are presenting with numerous chronic conditions. The prevalence of multimorbidity, the co-occurrence of two or more disorders, was 29.9% among adults and 80.2% among elderly aged 75 or older in Japan [24, 25]. The government and professional bodies need to counteract the limited popularity of family medicine. By increasing the number of family physicians and certified general practitioners, Japan will be in a better position to handle the multi- and complex-morbidity of patients and the geographic maldistribution of physicians in a quickly ageing society.

This study has the following limitations. First, the age of JPCA-certified family physicians was estimated. Therefore, we could not assert that physicians between the ages of 30 and 49 were the most appropriate comparison group. In addition, we could not adjust the factors related to physicians’ choice of practice location such as gender and birthplace because these data were not available. Moreover we cannot know whether each subject of this study is working in the private or public health sector due to the lack of such information in the original data-set. As mentioned earlier, the distribution of physicians in Japan is influenced by the cost of setting up a practice or clinic. Thus a future study should conduct a sub-analysis that compares the distribution of physicians in the private and public sectors.

## Conclusion

Article 25 of the Japanese Constitution states that everybody has a right to be healthy regardless of where they live or how much they earn. However, the geographic barrier to healthcare for rural residents has persisted despite half a century of financial and political investment by the government [4]. To improve the quality of care, streamline the provision of care, and especially to equalise the distribution of care, the Japanese government should increase the number of family physicians and certified general practitioners through national policies including offering financial incentives to medical students and physicians in training who hope to enter family practice.

## Data Availability

Conditions of the ethical approvals permit the cohort office (Department of Community-Based Medical Systems, Graduate School of Biomedical and Health Sciences, Hiroshima University) and the suboffice (Department of Community Medicine, Nagasaki University Graduate School of Biomedical Science) to share the aggregated data with stakeholders or researchers.

## Abbreviation

JPCA: The Japan Primary Care Association
